# Beneficial Effects of an Online Mindfulness-Based Intervention on Sleep Quality in Italian Poor Sleepers during the COVID-19 Pandemic: A Randomized Trial

**DOI:** 10.3390/ijerph20032724

**Published:** 2023-02-03

**Authors:** Teresa Fazia, Francesco Bubbico, Andrea Nova, Salvatore Bruno, Davide Iozzi, Beril Calgan, Giancarlo Caimi, Michele Terzaghi, Raffaele Manni, Luisa Bernardinelli

**Affiliations:** 1 Department of Brain and Behavioral Sciences, University of Pavia, 27100 Pavia, Italy; 2 Istituto di Psicosintesi, 20124 Milano, Italy; 3 Unit of Sleep Medicine and Epilepsy, IRCCS Mondino Foundation, 27100 Pavia, Italy

**Keywords:** COVID-19, emotion regulation, mindfulness, sleep quality, sleep stability, wellbeing

## Abstract

Sleep of inadequate quantity and quality is increasing in the present 24 h society, with a negative impact on physical and mental health. Mindfulness-based interventions (MBIs) generate a state of calm behavior that can reduce hyperactivity and improve sleep. We hypothesized that our specific MBI, administered online, may improve sleep quality and foster emotion regulation and mindfulness. The Pittsburgh Sleep Quality Index (PSQI), Sleep Condition Indicator (SCI), Arousal Predisposition Scale (APS), Ford Insomnia Response to Stress Test (FIRST), Sleep Hygiene Index (SHI) and Insomnia Severity Index (ISI) were used to measure sleep quality and stability. Emotion regulation and mindfulness were measured via the Emotion Regulation Questionnaire (ERQ) and Five Facet Mindfulness Questionnaire (FFMQ). Our MBI included 12 biweekly integral meditation (IM) classes, recorded IM training for individual practice, and dietary advice to promote sleep regulation. Fifty-six voluntary poor sleepers with a PSQI score of >5 were randomly allocated to treated (*n* = 28) and control (*n* = 28) groups. Linear mixed models were used to estimate the effectiveness of the intervention. Statistically significant results were observed in the FFMQ sub-domain *non-reactivity to inner experience* (β = 0.29 [0.06; −0.52], *p* = 0.01), PSQI (β = −1.93 [−3.43; −0.43], *p* = 0.01), SCI (β = 3.39 [0.66; 6.13], *p* = 0.02) and ISI (β = −3.50 [−5.86; −1.14], *p* = 0.004). These results confirm our hypothesis regarding the beneficial effects of our intervention on sleep quality.

## 1. Introduction

Sleep is an essential behavioral and neurological state. Hyperarousal, circadian dysrhythmia, homeostatic dysregulation and the activation of the sympathetic nervous system that generates a state of hyperactivity [1,2], contribute to sleep disturbances and disorders. 

Sleep problems have a relatively high incidence among the general population. Terzano et al. [3] reported a prevalence of insomnia among the Italian population of about 64%, and 44% of complained of diurnal disturbances as a consequence of their nocturnal disorder. A study carried out on 3970 Italians found that insomnia symptoms affected 27.6% of the sample, sleep dissatisfaction affected 10.1%, and 7% had a diagnosis of insomnia [4]. A more recent study on 3120 Italian adults revealed similar percentages [5]; specifically, sleep dissatisfaction was reported by 14.2% and insufficient sleep duration by 29.5% of the adults. In this scenario, the current COVID-19 pandemic emergency has profoundly changed people’s lifestyles, and in many cases has exacerbated sleep problems [6,7]. 

Pharmacological treatments for sleep disorders may come with considerable side effects, such as daytime sleepiness, poor cognitive performance and postural instability, particularly in the elderly [8]. Thus, non-pharmacological interventions are now considered as a first option or alternative treatment.

Psychological and behavioral interventions that incorporate many treatment components, including stimulus control, sleep restriction, relaxation, and cognitive therapy, can be considered effective alternative treatments [9,10]. Cognitive Behavioral Therapy, being a safe treatment with long-lasting beneficial effects, albeit not widely accessible, is considered as the first option to treat insomnia, recommended by the international guidelines of insomnia therapy [11]. 

Recently, in Euro-Western approaches and their medical point of view on human health, meditation techniques have also begun to emerge as alternative or additional treatment to the classic pharmacological and/or cognitive behavioral therapies to ameliorate sleep quality [12,13]. Meditation allows subjects to have greater mastery of the activities of the mind. It promotes a greater awareness of one’s mind and a state of non-judgmental acceptance, leading to better abilities of concentration and relaxation. Research shows its beneficial effects on emotional, psychological and physical levels in both clinical and non-clinical populations [14,15,16]. Mindfulness-based interventions (MBIs) that focus on breathing and directed attention, e.g., Mindfulness-Based Cognitive Therapy (MBCT) and Mindfulness-Based Stress Reduction (MBSR), improve sleep quality and insomnia symptoms both immediately after the intervention and in the follow-up, thus suggesting a crucial long-term effect [17]. 

Mindfulness, being a state of calm behavior, acts on the state of hyperactivity and improves self-regulation and attentional control thereby providing subjects with a strategy to improve their sleep [18]. Mindfulness can be used to raise awareness of the physical and mental states that are present when experiencing symptoms of insomnia. This would allow subjects to change their mental processes in response to these symptoms by promoting an adaptive and conscious attitude characterized by a more balanced assessment, by cognitive flexibility and equanimity [19]. Maintaining a conscious attitude allows the sleep-related arousal state to decrease and normal sleep patterns to re-emerge [18]. 

Furthermore, healthy eating habits, balancing macronutrients and the necessary intake of vitamins and minerals without overconsuming unhealthy foods, and adopting specific diets, particularly based on the consumption of tryptophan-containing food (e.g., legumes, dairy products, whole grain, etc.), may improve the quality and quantity of sleep [20,21]. In addition, short sleep duration was found to be associated with obesity [22] and higher total calories [23,24]. In particular, in the study by Grandner et al. [23], higher absolute protein, carbohydrate, sugar, and total fat intake, and lower intake of dietary fiber were found in short sleepers (5–6 h) rather than normal sleepers (7–8 h). For these reasons, it is conceivable that providing instructions for a healthy diet with tryptophan-containing food can act synergistically with other treatments to improve sleep quality.

However, although when investigating MBIs, promising results have been obtained, additional well-planned studies are still needed, especially for the estimation of the dose–response effect. 

Here, we assessed the effect of a short, online MBI on the sleep pattern and quality of a sample of Italians poor sleepers. Specifically, our MBI comprised: (i) 12 twice-weekly online integral meditation classes (IM), a mindfulness-based technique tested here for the first time on poor sleepers; (ii) a recorded IM class for individual practice; and (iii) dietary advice to promote sleep regulation. Our research hypothesis was that this intervention has a beneficial effect on the mental life of individuals which in turn improves sleep patterns and therefore ameliorates the symptoms of insomnia. To this aim, we estimated the effectiveness of the proposed intervention on six outcomes related to sleep patterns and quality using six self-reported questionnaires, i.e., the Pittsburgh Sleep Quality Index (PSQI), Sleep Condition Indicator (SCI), Arousal Predisposition Scale (APS), Ford Insomnia Response to Stress Test (FIRST), Sleep Hygiene Index (SHI) and Insomnia Severity Index (ISI). Furthermore, we hypothesized that our intervention fosters emotion regulation and mindfulness as measured by the Emotion Regulation Questionnaire (ERQ) and Five Facet Mindfulness Questionnaire (FFMQ), respectively. In addition, we also tried to investigate a possible dose–response effect on all the studied outcomes.

## 2. Materials and Methods

### 2.1. Participants

Subjects were recruited via digital (social networks and mailing) means in September 2020 and were asked to complete the PSQI to select participants with low sleep quality (target population). The inclusion criteria were: (i) having a PSQI score of >5, indicating impaired sleep quality, and (ii) not suffering from severe anxiety or depression, severe mental illness (e.g., hypomania or psychotic episodes), or any other diagnosed mental or physical health condition at the time of recruitment. This second criterion was checked by directly asking the participant about their condition.

Eligible participants signed an informed consent and privacy policy and were randomly assigned to an intervention or control group, using a simple randomization procedure with a 1:1 allocation ratio, by using the R package randomize. Subjects in the passive control group, for ethical reasons, were offered the same intervention as the treatment group once the study was completed. 

The study was registered retrospectively in the ISRCTN registry (ISRCTN39530091). 

### 2.2. Intervention

The intervention consisted of 12 mindfulness-based IM classes given twice a week from October to December 2020. Each class lasted approximately 60 min and was delivered on the Zoom video conferencing platform [25]. Briefly, our IM training, which represents the core element of our intervention, involves: (i) developing awareness of the body and mind in terms of improving the ability to generate relaxation and mental peace; (ii) stabilizing the mind to stop ruminations and perennial inner chatter; (iii) refining the ability to hear and recognize the dynamics between emotions and thought; and (iv) enhancing the balance between openness to others and attention to oneself that in turn promotes psycho-physical well-being in general. IM simultaneously uses breathing, focused attention, the release of physical tensions, thoughts and sensations through internal senses and imagery. This enables rapid relaxation and a deep physical, energetic, and spiritual well-being. IM has a demonstrated efficacy in the non-clinical general population as reported in previous studies [26,27,28]. Here, for the first time, we tested its efficacy on poor sleepers, so the IM protocol was slightly adjusted to meet the needs of the target population, placing a particular emphasis on self-body and emotional listening, and thought awareness, in order to differentiate positive and negative thoughts. An expert meditator (S.B.) developed and administered the training.

Participants also received a 25 min audio recording to practice IM daily before going to sleep, to release physical and emotional tension through focused breathing and body-part relaxation. In addition, they received general non-mandatory dietary advice and recommendations from a nutritionist aimed at promoting healthy sleep. In particular, the consumption of tryptophan-containing food (e.g., legumes, dairy products, whole grain, etc.) was recommended [20]. The capacity of tryptophan to improve sleep resides in its conversion into serotine, and this largely depends on its ability to cross the blood–brain barrier, which in turn seems to be favored by the consumption of high-carbohydrate meals [29]. In fact, the increased plasma glucose leads to insulin secretion, thus removing circulating large neutral amino acids in favor of tryptophan, which in this way can cross the blood–brain barrier and be converted into serotonin. Subjects characterized by self-reported high levels of inflammations were also recommended to reduce the consumption of histamine-rich food (e.g., tomatoes, spinach, eggplant, parmesan, blue cheese, red wine, etc.). Furthermore, scientific evidence suggests histamine in the central nervous system as a player in the regulation of sleep–wakefulness through its receptors, especially H1 and H3 [30], so that the H1 receptor antagonist is often used for the treatment of insomnia, even if with potential side effects.

### 2.3. Psychological and Sleep Metrics

Each participant in both groups at both t0 (before the start of the study) and t1 (at the end of the study) filled the validated Italian versions of eight self-report questionnaires: two measuring psychological factors (ERQ and FFMQ) and six measuring the parameters of sleep quantity, global quality, and continuity (PSQI, APS, SCI, FIRST, ISI, SHI). Details are provided in the Supplementary Materials. Sociodemographic information was also collected from each participant through a background questionnaire administered at t0 only. All questionnaires were completed online via Google Forms.

At the end of the study, participants in the treatment group were also asked to evaluate their adherence to the dietary advice on a scale from 1 (“I have not followed the dietary advice”) to 10 (“I followed the dietary advice at every meal”).

### 2.4. Statistical Analysis

This was a two-group pre–post experimental design, in which participants were randomly assigned to the intervention or the control group. For each group, outcome variables were collected at t0 and t1 and subjects who did not fill in the proposed questionnaires at both time points were excluded from the analysis. Questionnaires were scored following the provided guidelines, and, for each questionnaire, internal consistency was assessed via Cronbach’s α coefficient using the Cronbach function in the psy R package (version 1.2) [31]. 

Differences between the baseline characteristics of the groups, collected through the background questionnaire, were investigated for each continuous variable by using *t*-test or Wilcoxon test, and for each categorical variables by using chi-squared or Fisher’s exact test. The Hedges’ Effect size statistic was calculated for each variable by using the cohen.d function in the effsize R package (version 0.8.1).

Linear Mixed Effect (LME) models [32] were applied to evaluate the pre–post treatment changes on each outcome using the lme function in the nlme R package (version 3.1). A random intercept for subjects in the form of 1|subject was used to adjust the models for intra-subject variability, produced by the two repeated measurements carried out on the same subject. To test our research hypotheses regarding the beneficial effect of our intervention on ameliorating sleep problems and on fostering emotion regulation and mindfulness, we investigated the six sleep-related outcomes and the two psychological indicators. For each outcome, the coefficient of the interaction between time and treatment indicating how much more the intervention group improved over time with respect to the investigated endpoints, compared to the control group over the same period, was estimated. Standardized coefficients were also calculated by using the parameters R package (version 0.20.1). All models were adjusted for sex, age, previous meditation experience and the consumption of drugs for sleep disturbance. For each model, the normality of residuals was assessed graphically through Q-Q plots and via the Shapiro–Wilk test. In the case of the non-normality of residuals, empirical bootstrap with 5000 bootstrapped replicates was applied to estimate non-parametric 95% C.I.s and *p*-values based on distribution’s quantiles [33]. The Benjamini–Hochberg correction, fixing a false discovery rate (FDR) at alpha < 0.05, was used to account for multiple comparisons [34] (using the p.adjust function in the stats R package (version 4.2.2)). Nevertheless, given the explanatory nature of our study, the limited number of hypotheses tested, and the need to avoid missing important findings, we also discussed the results with an unadjusted *p*-value of <0.05 [35].

For the statistically significant score change, considering an unadjusted *p*-value of <0.05, a clinical significance analysis was conducted by determining the percentage of subjects that had improved or recovered, moving from the clinical to the functional population. This analysis was implemented using the clinicalsignificance R package (version 1.2.0).

A linear model of the number of IM classes attended by each participant on each post–pre difference in questionnaire score (Δ) was also fitted to test the possible dose–response effect using the number of meditation sessions attended (in days) as an explanatory variable, controlling for sex, age, previous meditation experience and consumption of drugs for sleep disturbance treatment. 

Descriptive statistics are reported as the mean ± standard deviation (SD). All analysis were performed using R 3.5.1 software [36].

## 3. Results

**Figure 1:**
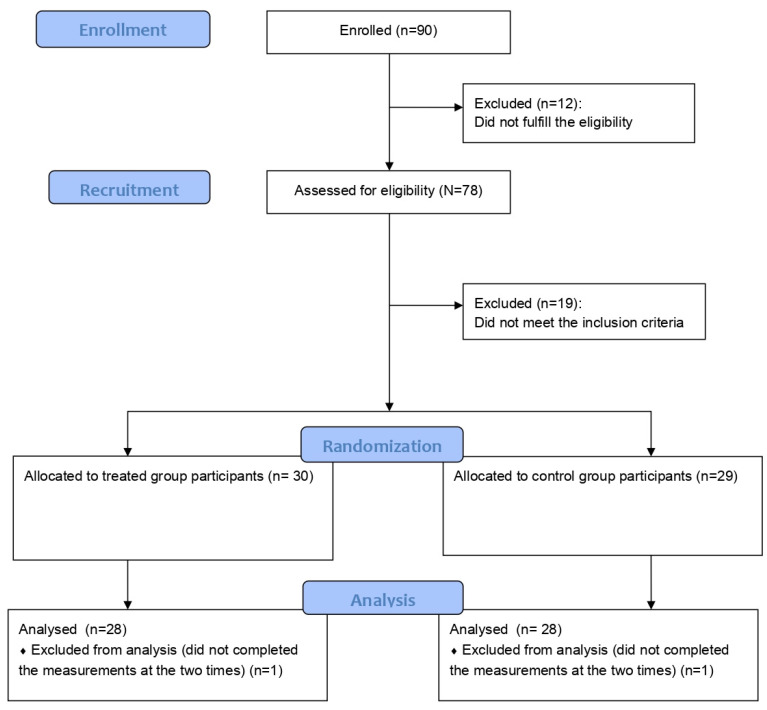
Participant CONSORT flow diagram.

Ninety subjects showed interest in participating in the study and received the eligibility questionnaire: 78 filled in the questionnaire but only 59 fulfilled the inclusion criteria and were randomly allocated to the treated (*n* = 30) or control (*n* = 29) group. Of these 59 eligible subjects, two in the treated and one in the control group did not fill in all the questionnaires so were excluded from the analysis. This led to a final total sample of 56 subjects, 28 per group, 46 females and 10 males, with a mean age of 53.7 (SD = 11.6) (see Figure 1). 

The baseline (t0) characteristics of the participants for the two groups are shown separately in Table 1. No statistically significant differences were observed between the two groups for the baseline parameters. In Table 2, mean, SD and internal consistency for each questionnaire and subscale in the two groups at both time points are reported. In Supplementary Figure S1, the means and SD for each outcome were plotted by time and by group.

It is worth noting that at t0, 39 subjects (19 controls and 20 treated) had an SCI questionnaire score of <16, indicating an insomnia condition; all of them also reported a chronic insomnia condition (persisting for at least three months) as measured by the 8th item of the SCI questionnaire. After the intervention, 16 subjects (12 controls and only four treated) had an SCI questionnaire score of <16. As regards PSQI, at t0, the whole sample had a score of ≥5, indicating poor sleep quality, while at t1, three subjects in the control group and seven subjects in the treatment group had a score of <5, indicating that they no longer had a poor sleep quality. Moreover, before the intervention, a total of 13 subjects reported sleeping less than five hours a day (seven controls and six treated), while at t1, a total of eight subjects reported sleeping less than five hours a day (six controls and two treated), as measured by Component 3, i.e., sleep latency, of the PSQI.

The mean number of IM classes attended by the 38 participants was 8.9 over a total of 12 classes with 78% of the participants attending at least seven classes. This indicates good participation by our subjects in the intervention proposed. Thus, IM class participation showed the applicability of our study to those who wanted to improve sleep quality. 

As for the dietary advice in the intervention group, 15 subjects reported a low adherence, eight a medium adherence, and five a high adherence to the dietary program.

Data were analyzed following an intention-to-treat (ITT) approach and LME models were used to estimate the effectiveness of the intervention on the investigated outcomes. The results, as reported in Table 3, both considering adjusted and unadjusted *p*-value, support the hypothesis of the beneficial effect of our intervention on the sub-domain of FFMQ *non-reactivity to inner experience* (β = 0.29 [0.06; 0.52], unadjusted *p* = 0.01 and adjusted *p* = 0.04), on PSQI (β = −1.95 [−3.43; −0.42], unadjusted *p* = 0.01 and adjusted *p* = 0.04), on PSQI Component 1 (β = −0.53 [−0.85; −0.22], unadjusted *p* = 0.001 and adjusted *p* = 0.02), on PSQI Component 3 (β = −0.54 [−0.92; −0.15], unadjusted *p* = 0.007 and adjusted *p* = 0.04) and on ISI (β = −3.50 [−5.86; −1.14], unadjusted *p* = 0.004 and adjusted *p* = 0.04). As for SCI, the result was not statistically significant after a multiple testing correction (adjusted *p* = 0.07) even if, as stated in the statistical methods section, we still discussed this interesting result (β = 3.39 [0.66; 6.13], unadjusted *p* = 0.02). The same was true for PSQI Component 7 (β = −0.39 [−0.76; −0.03], adjusted *p* = 0.09). No beneficial effect was observed on the remaining scales. Higher scores on the FFMQ and SCI indicate, respectively, a greater dispositional mindfulness and fewer symptoms of insomnia, while a lower score in ISI indicated a lower severity of insomnia symptoms. Lower scores in PSQI, as well as in its subscales, indicated a better sleep quality over the last month.

A subsequent clinical significance analysis was performed on PSQI total score, PSQI Components 1, 4, 7, ISI and SCI by using the mean and standard deviation of the functional population derived by [37,38,39]. This analysis showed that: (*i*) 36% of the treated vs. 25% of the controls improved in the FFMQ *non-reactivity to inner experience* scores; (*ii*) 71% of the treated vs. 57% of the controls improved and 25% of the treated vs. 11% of the control recovered according to the PSQI total score; (*iii*) 57% of the treated vs. 18% of the controls improved and 4% of the treated vs. 0% of the controls recovered according to PSQI Component 1; (*iv*) 39% of the treated vs. 18% of the controls improved and 21% of the treated vs. 14% of the controls recovered according to PSQI Component 3; (*v*) 18% of the treated vs. 21% of the controls improved and 32% of the treated vs. 7% of the controls recovered according to PSQI Component 7; (*vi*) 54% of the treated vs. 64% of the controls improved and 18% of the treated vs. 0% of the controls recovered according to the SCI scores; and (*vii*) 21% of the treated vs. 29% of the controls improved and 43% of the treated vs. 14% of the controls recovered according to the ISI scores.

Furthermore, as regards the dose–response effect of the number of attended IM sessions on the pre–post differences in the questionnaire scores, no statistically significant dose–response effect was observed (see Supplementary Table S1). This result must be interpreted with caution as the amount of time the participants engaged in individual practice of meditation was not recorded. For this reason, it was difficult to estimate a dose–response effect based only on IM class attendance.

## 4. Discussion

Sleep disorders, as characterized by poor sleep quantity and/or quality, have a high prevalence in the general population and are associated with an increased risk of developing mental, metabolic, and cardiovascular diseases, thus representing a public health issue [40,41]. For this reason, there is an urgent need to find easily accessible, effective, safe alternative treatments.

In our study, we tested the beneficial effect of a short online MBI on sleep quality, quantity, and continuity, and on emotional regulation and mindfulness in poor sleepers. We detected a beneficial effect of our intervention in improving sleep quality and in decreasing insomnia symptoms and severity, as indicated by the statistically significant results obtained in the global score of PSQI (β = −1.93 [−3.43; −0.43], *p* = 0.01), in SCI (β = 3.39 [0.66; 6.13], *p* = 0.02) and in ISI (β = −3.50 [−5.86; −1.14], *p* = 0.004). These results indicate that our intervention represents an effective tool in promoting healthy sleep components in subjects with poor sleep quality and support the current literature which indicates how MBIs improve sleep quality in people with sleep disturbances [12,13].

It is important to note that before the intervention, 68% of the participants in the control group and 71% of the participants in the intervention group reported an insomnia condition. This rate decreased to 57% and 14%, respectively, after the intervention. Furthermore, 10% of the participants in the control group and 25% of the participants in the treated group reported no longer having poor sleep quality.

Without considering the psychological scales (ERQ and FFMQ), the smallest effect size we found was on the use of sleep medication (PSQI component); followed by the effect size on the SHI and FIRST scales. The largest effect size was found on subjective sleep quality (PSQI Component 1), followed by the ISI scale. As regards PSQI, our results are in line with the effects reported for educational, behavioral and/or cognitive treatments for insomnia [9], while we found a slightly smaller effect size for ISI. The effect sizes found in our research are overall larger than those found in other studies that tested the efficacy of mindfulness meditation on sleep quality compared to specific or non-specific active control groups [12], probably because in these studies the control group interventions might have had an impact on sleep health in these studies. The effect size found in other studies which used waiting-list control or attention control groups [13] ranged from small to large, similarly to ours, despite the fact that we found a medium effect size for PSQI compared to the large effect size reported in other studies.

In the interpretation of our results, it is helpful to consider that this study took place during a period when COVID-19 was still very widespread in Italy, and as shown in a study conducted on 2291 Italian, 57.1% experienced poor sleep quality, 32.1% high levels of generalized anxiety symptoms, and 41.8% psychological distress [42]; another study also found widespread pre-sleep arousal [43], thus demonstrating how the participants’ sleep quality was strongly affected by the COVID-19 situation in Italy.

We found no changes in predisposition to arousability (as measured by APS), nor in the sleep reactivity trait (as measured by FIRST). These results might be explained by the fact that these are probably too stable traits to change after such a short intervention. Interestingly, in our study, 40 out of 56 subjects yielded a FIRST score of ≥18 at t0, thus indicating a high risk of insomnia [44]. 

As for sleep hygiene behaviors, measured by SHI, we found no changes either on the whole scale or on single items. We expected that the change to a more mindful mindset would result in the improvement of some dysregulated behaviors. Since the participants showed only a small change or no change in their level of different mindfulness domains, we can assume that the benefits of the intervention led only to more immediate effects (i.e., stress relief, relaxation) that helped the participants to improve their sleep without affecting their mindset in a significant way. Another possible explanation is the high average age of participants. Spontaneous changes in habitual rooted behavior are more difficult. Although the dietary advice was a non-mandatory part of our intervention, we registered a low–medium adherence to the diet in most of the participants. As confirmed by SHI, the participants showed difficulty in actively regulating or changing their eating behaviors, even when they knew it could improve their sleep quality.

Whilst we expected our intervention to produce noticeable changes in emotional regulation strategies and dispositional mindfulness, we did not find significant effects on them, except for on a sub-component of dispositional mindfulness, which is non-reactivity to inner experience. A possible interpretation is that our intervention was only effective in helping participants reach healthier sleep by providing them with a practical tool to auto-regulate their biological and psychological systems. This means that our intervention worked efficiently on the compelling evident needs of the participants without being able to produce a deeper shift in their emotional regulation strategies and their overall mindfulness level. Such a shift might require more consistent practice.

The limitations of our study were: (i) the results may only be applicable to people who are willing to meditate and interested in the theme of meditation given the nature of our intervention, i.e., the obviously voluntary-based enrolment; (ii) the sole use of self-report measures; and (iii) the absence of monitoring of the individual practice of meditation with subsequent difficulties in the estimation of a dose–response effect.

## 5. Conclusions

In conclusion, this study shows the effectiveness of a low-cost and easy-to-access online MBI in improving sleep quality and reducing insomnia symptoms and severity among poor sleepers. Hence, it is a practice that it is worthwhile adopting to also improve one’s quality of life. 

## Figures and Tables

**Table 1:** Baseline characteristics of the analyzed sample (treated = 28, control = 28).

Variables	Mean (SD) Controls	Mean (SD) Treated	*p*-Value
***Age***	53.96 (13.31)	53.46 (10.04)	0.87
	**N (%) controls**	**N (%) treated**	
***Sex***			
Male	5 (18%%)	5 (18%)	1
Female	23 (82%)	23 (82%)	
***Nationality***			
Italian	26 (93%)	28 (100%)	0.49
Non-Italian	2 (7%)	0 (0%)	
***Marital status***			
Cohabitant/married	11 (39%)	16 (57%)	0.36
Unmarried/single	8 (29%)	4 (14%)	
Separated/Divorced	8 (29%)	8 (29%)	
Widowed	1 (4%)	0 (0%)	
***Number of children***			
0	10 (36%)	11 (39%)	0.43
1	6 (21%)	8 (28%)	
2	9 (32%)	9 (32%)	
≥3	3 (11%)	0 (0%)	
***Dependent children/family members***			
No	18 (64%)	18 (64%)	1
Yes	10 (36%)	10 (36%)	
***Unpaid loans***			
No	21 (75%)	22 (79%)	1
Yes	7 (25%)	6 (21%)	
***Education***			
Middle school	1 (4%)	1 (4%)	1
High school	11(39%)	10 (36%)	
Degree	13 46(%)	14 (50%)	
Post-graduate course (e.g., PhD)	3 (11%)	3 (11%)	
***Job***			
Public or private employee	15 (54%)	16 (57%)	0.35
Freelance (e.g., lawyer, doctor etc.)	4 (14%)	3 (11%)	
Student	0 (0%)	1 (4%)	
Unemployed or looking for a job	3 (11%)	0 (0%)	
Housewife	0 (0%)	2 (7%)	
Retired	6 (21%)	6 (21%)	
***Type of employment agreement***			
Undetermined term	13 (46%)	15 (54%)	0.80
Fixed term	4 (14%)	2 (7%)	
Not applicable	11 (39%)	11 (39%)	
***Employee satisfaction***			
No	12 (43%)	7 (25%)	0.26
Yes	16 (57%)	21 (75%)	
***Sport***			
No	5 (18%)	8 (29%)	0.53
Yes	23 (82%)	20 (71%)	
***Smoker***			
Yes	4 (14%)	4 (14%)	1
No	24 (86%)	24 (86%)	
***Knowledge about meditation***			
Clear idea	19 (68%)	16 (57%)	0.40
Vague idea	8 (29%)	12 (43%)	
Just heard of	1 (4%)	0 (0%)	
***Previous meditation experience***			
Yes	22 (79%)	24 (86%)	0.73
No	6 (21%)	4 (14%)	
***Religious***			
No	13 (46%)	16 (57%)	0.59
Yes	15 (54%)	12 (43%)	
***Number of books read in a year***			
0–1	4 (14%)	2 (7%)	0.52
2–3	6 (21%)	4 (14%)	
>3	18 (64%)	22 (79%)	
***Member of a cultural/sportive association***			
No	20 (71%)	17 (61%)	0.57
Yes	8 (29%)	11 (39%)	
***Diet***			
Mediterranean	22 (79%)	24 (86%)	0.69
Vegetarian	2 (7%)	1 (4%)	
Vegan	3 (11%)	1 (4%)	
Macrobiotic	1 (4%)	2 (7%)	
***Disease/disability***			
No	20 (71%)	15 (54%)	0.27
Yes	8 (29%)	13 (46%)	
***Addiction***			
No	27 (96%)	25 (89%)	0.61
Yes	1 (4%)	3 (11%)	
***Have you ever gone to a psychologist***			
No	10 (36%)	11 (39%)	1
Yes	18 (64%)	17 (61%)	
***Drug for insomnia***			
No	21 (75%)	17 (61%)	0.39
Yes	7 (25%)	11 (39%)	

**Table 2:** Mean, SD, internal consistency, and Hedges’ effect size for each questionnaire and subscale in the two groups (controls and treated) at both time points (t_0_ and t_1_).

Questionnaire	Mean (SD) Controls t0	Mean (SD) Treated t0	Mean (SD) Controls t1	Mean (SD) Treated t1	Internal Consistency t0	Internal Consistency t1	Effect Size (Hedges’ g)
***ERQ***							
Reappraisal	4.97 (1.09)	5.09 (1.20)	5.05 (1.13)	4.88 (1.26)	0.89	0.91	−0.31 (small)
Suppression	3.68 (1.05)	3.39 (1.36)	3.52 (1.25)	3.60 (1.04)	0.70	0.61	0.32 (small)
***FFMQ***							
All Items	3.26 (0.52)	3.18 (0.51)	3.25 (0.44)	3.30 (0.41)	0.93	0.88	0.50 (medium)
Observing	3.41 (0.84)	3.49 (0.68)	3.37 (0.79)	3.49 (0.67)	0.85	0.83	0.07 (negligible)
Describing	3.59 (0.81)	3.50 (0.77)	3.57 (0.73)	3.64 (0.75)	0.92	0.91	0.42 (small)
Acting with awareness	3.22 (0.78)	3.01 (0.73)	3.27 (0.77)	3.15 (0.63)	0.91	0.88	0.17 (negligible)
Non-judging of inner experience	3.18 (0.92)	3.18 (0.88)	3.22 (0.79)	3.28 (0.84)	0.93	0.91	0.09 (negligible)
Non-reactivity to inner experience	2.83 (0.65)	2.68 (0.59)	2.73 (0.73)	2.88 (0.58)	0.81	0.83	0.66 (medium)
***PSQI***							
All Items	11.61 (3.71)	10.64(3.03)	9.86 (3.97)	6.96 (3.18)	0.60	0.70	−0.67 (medium)
Subjective sleep quality (Component 1)	1.86 (0.65)	1.82 (0.72)	1.75 (0.58)	1.18 (0.47)			−0.89 (large)
Sleep latency (Component 2)	2.14 (1.04)	1.46 (0.79)	1.50 (1.14)	1.04 (0.79)			0.27 (small)
Sleep duration (Component 3)	1.79 (0.83)	1.96 (0.69)	1.61 (0.99)	1.25 (0.89)			−0.74 (medium)
Sleep efficiency (Component 4)	1.46 (1.26)	1.29 (0.98)	1.54 (1.35)	0.82 (1.06)			−0.45 (small)
Sleep disturbance (Component 5)	1.64 (0.68)	1.57 (0.50)	1.50 (0.64)	1.18 (0.48)			−0.40 (small)
Use of sleep medication (Component 6)	1.29 (1.18)	1.32 (1.36)	0.71 (1.08)	0.86 (1.27)			0.11 (negligible)
Daytime dysfunction (Component 7)	1.43 (0.63)	1.21 (0.63)	1.25 (0.64)	0.64 (0.56)			−0.57 (medium)
***APS***							
All Items	38.96 (6.24)	37.57 (6.62)	39.00 (7.59)	38.39 (6.05)	0.85	0.86	0.17 (negligible)
***SCI***							
All Items	13.39 (6.78)	13.82 (5.68)	16.46 (5.56)	20.28 (5.01)	0.81	0.78	0.66 (medium)
***FIRST***							
All Items	22.03 (6.86)	23.07 (5.45)	22.71 (6.51)	23.21 (6.12)	0.90	0.89	−0.12 (negligible)
***ISI***							
All Items	11.75 (4.35)	11.64 (4.33)	11.32 (5.00)	7.71 (3.89)	0.82	0.83	−0.78 (medium)
***SHI***							
All Items	29.61 (5.76)	28.11 (4.72)	28.57 (5.80)	26.61 (5.29)	0.61	0.64	−0.12 (negligible)

**Table 3:** Between-group differences obtained using a linear mixed model. For each questionnaire and subscale, both standardized and non-standardized β coefficients of time*group interaction with their 95% CI and *p*-value are reported.

Questionnaire	β Time*Group [95%CI]	Standardized β Time*Group [95%CI]	Unadjusted *p*-Value	Adjusted *p*-Value
***ERQ***				
Reappraisal	−0.29 [−0.90; 0.36]	−0.13 [−0.35; 0.09]	0.34	0.51
Suppression	0.37 [−0.25; 0.98]	0.16 [−0.11; 0.42]	0.24	0.42
***FFMQ***				
All Items	0.12 [−0.01; 0.26]	0.13 [−0.01; 0.27]	0.06	0.16
Observing	0.03 [−0.22; 0.29]	0.02 [−0.15; 0.20]	0.78	0.78
Describing	0.16 [−0.04; 0.37]	0.11 [−0.03; 0.25]	0.11	0.23
Acting with awareness	0.08 [−0.17; 0.34]	0.06 [−0.12; 0.24]	0.51	0.68
Non-judging of inner experience	0.07 [−0.30; 0.44]	0.04 [−0.18; 0.26]	0.72	0.76
Non-reactivity to inner experience	0.29 [0.06; 0.52]	0.23 [0.04; 0.41]	**0.01**	**0.04**
***PSQI***				
All Items	−1.93 [−3.43; −0.42]	−0.25 [−0.45; −0.05]	**0.01**	**0.04**
Subjective sleep quality	−0.53 [−0.85; −0.22]	−0.40 [−0.64; −0.16]	**0.001**	**0.02**
Sleep latency	0.21 [−0.21; 0.63]	0.11 [−0.10; 0.31]	0.31	0.50
Sleep duration	−0.54 [−0.92; −0.15]	−0.30 [−0.52; −0.09]	**0.007**	**0.04**
Sleep efficiency	−0.54 [−1.16; 0.09]	−0.23 [−0.49; 0.04]	0.09	0.21
Sleep disturbance	−0.25 [−0.58; 0.08]	−0.21 [−0.48; 0.06]	0.13	0.25
Use of sleep medication	0.11 [−0.39; 0.60]	0.04 [−0.16; 0.24]	0.66	0.73
Daytime dysfunction	−0.39 [−0.76; −0.03]	−0.29 [−0.56; −0.02]	**0.03**	0.09
***APS***				
All Items	0.79 [−1.68; 3.25]	0.06 [−0.13; 0.25]	0.52	0.68
***SCI***				
All Items	3.39 [0.66; 6.13]	0.27 [0.05; 0.49]	**0.02**	0.07
***FIRST***				
All Items	−0.54 [−2.97; 1.90]	−0.04 [−0.24; 0.15]	0.66	0.73
***ISI***				
All Items	−3.50 [−5.86; −1.14]	−0.38 [−0.63; −0.12]	**0.004**	**0.04**
***SHI***				
All Items	−0.46 [−2.44; 1.52]	−0.04 [−0.23; 0.14]	0.64	0.73

*p*-values ≤ 0.05 are considered statistically significant. All models are adjusted for sex, age, previous meditation experience and use of drugs for insomnia.

## Data Availability

All datasets presented in this study are included in the Supplementary Material.

## Supplementary Materials

The following supporting information can be downloaded at: https://www.mdpi.com/article/10.3390/ijerph20032724/s1. References [45,46,47,48,49,50,51,52] are cited in the Supplementary Materials.
