# Supplementary material for: Beneficial Effects of an Online Mindfulness-Based Intervention on Sleep Quality in Italian Poor Sleepers during the COVID-19 Pandemic: A Randomized Trial
Source: Int J Environ Res Public Health. 2023 Feb 3;20(3):2724. doi: 10.3390/ijerph20032724 (PMC9914977; doi:10.3390/ijerph20032724)
Supplement: Supplementary file 1 [file ijerph-20-02724-s001.zip › Suppplementary Material.pdf]

Beneficial effects of an online mindfulness-based intervention on sleep quality in Italian poor-sleepers during the COVID-19 pandemic: a randomized trial

Supplementary Material

Each participant in both groups at both t0 (before the start of the study) and t1 (at the end of the study) filled in eight self-report questionnaires; two measuring psychological factors (ERQ and FFMQ) and six measuring parameters of sleep quantity, global quality and continuity (PSQI, APS, SCI, FIRST, ISI, SHI).

As for the sleep-related outcomes:

The PSQI questionnaire comprises 19 questions exploring seven different components of sleep quality over a month: sleep duration, sleep disturbance, sleep latency, daytime dysfunction due to sleepiness, sleep efficiency, overall sleep quality, and sleep medication used, plus five questions rated by the bed partner or roommate (if one is available). Each item is rated from 0 to 3. PSQI scores > 5 and ≤ 5 indicated poor and good sleep quality, respectively. Sleep disorders are determined when PSQI>7[1].

The SCI is an eight-item rating scale that was developed to screen for insomnia disorder based on the Diagnostic and Statistical Manual of Mental Disorders (DSM)-5 criteria. The SCI comprises two items on sleep continuity (item 1: getting to sleep; item 2: remaining asleep), two items on sleep satisfaction/dissatisfaction (item 4: sleep quality; item 7: troubled or not), two items on severity (item 3: nights per week; item 8: duration of problem), two items on attributed daytime consequences of poor sleep (item 5: effects on mood, energy or relationships (personal functioning); item 6: effects on concentration, productivity, or ability to stay awake (daytime performance))[2] Each item is rated on a scale from 0 to 4. The total score can range from 0 to 32, with higher values indicative of better sleep.

The ISI is a seven-item questionnaire assessing the nature, severity, and impact of insomnia. The usual recall period is the “last month” and the dimensions evaluated are: severity of sleep onset, sleep maintenance, and early morning awakening problems, sleep dissatisfaction, interference of sleep difficulties with daytime functioning, noticeability of sleep problems by others, and distress caused by the sleep difficulties. A five-

point Likert scale is used to rate each item (0=no problem; 4=very severe problem) and total scores range from 0 to 28. Between 22 and 28 points indicate a possible clinical insomnia problem[3].

The SHI is a 13-item index developed to assess the presence of sleep hygiene behaviors. Participants are asked to show how frequently they engaged in specific behaviors (always, frequently, sometimes, rarely, never). Each item is rated on a five-point Likert scale (ranging from 0=never to 4=always). The total scores ranged from 0 to 52, with higher scores revealing more maladaptive sleep hygiene status[4].

The FIRST is a nine-item scale used to assess an individual's likelihood of experiencing sleep difficulties in response to common stressful situations. Each item is self-rated on a four-point Likert scale and summed to yield a score between 9 and 36, higher scores indicate higher levels of sleep reactivity[5].

The APS is a 12-item scale that measures arousability. Each item is in the format of a self-description (e.g., "I get excited easily"). Subjects can select one of five responses: "never", "seldom", "occasionally", "frequently" or "always". Six specific indexes frequently associated with insomnia are assessed: (1) delayed sleep onset latency, (2) frequent night awakenings, (3) frequent nightmares, (4) dormital restlessness, (5) early morning awakenings, and (6) subjective feelings of tiredness upon arising[6].

As for the psychological outcomes:

The ERQ is a 10-item questionnaire that consists of two scales corresponding to two different emotion regulation strategies: cognitive reappraisal (six items) and expressive suppression (four items). The 10 items are rated on a seven-point Likert scale from strongly disagree to strongly agree[7].

The FFMQ is a 39-item multidimensional assessment tool designed to measure a person's level of mindfulness. In particular, it is aimed to measure five interrelated components of mindfulness, which are: (1) observing (three items), (2) describing (three items), (3) acting with awareness (three items), (4) non-judging of inner experiences (three items), (5) non-reactivity to inner experience (three items). A higher score in the FFMQ full-scale as well as in its subscales reflects a higher level of mindfulness[8]. The questionnaire has shown good psychometric properties both in the English and Italian versions, which show a similar factorial structure to the original. A higher score after an intervention reflects an improved level of mindfulness.

References

[1] Buysse DJ, Reynolds CF, Monk TH, Berman SR, Kupfer DJ. The Pittsburgh Sleep Quality Index: a new instrument for psychiatric practice and research. Psychiatry Res 1989;28:193–213. https://doi.org/10.1016/0165-1781(89)90047-4.

[2] Espie CA, Kyle SD, Hames P, Gardani M, Fleming L, Cape J. The Sleep Condition Indicator: a clinical screening tool to evaluate insomnia disorder. BMJ Open 2014;4. https://doi.org/10.1136/BMJOPEN-2013-004183.

[3] Morin CM, Belleville G, Bélanger L, Ivers H. The Insomnia Severity Index: psychometric indicators to detect insomnia cases and evaluate treatment response. Sleep 2011;34:601–8. https://doi.org/10.1093/SLEEP/34.5.601.

[4] Mastin DF, Bryson J, Corwyn R. Assessment of sleep hygiene using the Sleep Hygiene Index. J Behav Med 2006;29:223–7. https://doi.org/10.1007/S10865-006-9047-6.

[5] Drake C, Richardson G, Roehrs T, Scofield H, Roth T. Vulnerability to stress-related sleep disturbance and hyperarousal. Sleep 2004;27:285–91. https://doi.org/10.1093/SLEEP/27.2.285.

[6] Coren S, Mah KB. Prediction of physiological arousability: a validation of the Arousal Predisposition Scale. Behav Res Ther 1993;31:215–9. https://doi.org/10.1016/0005-7967(93)90076-7.

[7] Gross JJ, John OP. Individual differences in two emotion regulation processes: implications for affect, relationships, and well-being. J Pers Soc Psychol 2003;85:348–62.

[8] Baer RA, Smith GT, Hopkins J, Krietemeyer J, Toney L. Using Self-Report Assessment Methods to Explore Facets of Mindfulness. Assessment 2006;13:27–45. https://doi.org/10.1177/1073191105283504.

Supplementary Table

Supplementary Table S1. Linear model for the effect of the number of meditation sessions attended in the treatment group on each questionnaire and subscale (pre-post difference). β coefficient of days of meditation (β days) with its 95% CI, and unadjusted *p*-value are reported.

Questionnaire	β Days [95% CI]	Unadjusted p-value
ERQ		
Reappraisal	0.01 [-0.12;0.15]	0.86
Suppression	0.002 [-0.13;0.13]	0.98
FFMQ		
All Items	0.01 [-0.02;0.04]	0.65
Observing	-0.001 [-0.04;0.04]	0.96
Describing	-0.03 [-0.07;0.004]	0.08
Acting with awareness	0.03 [-0.01;0.08]	0.14
Non-judging of inner experience	0.02 [-0.07;0.10]	0.72
Non-reactivity to inner experience	0.02 [-0.04;0.07]	0.50
PSQI		
All Items	0.14 [-0.17;0.46]	0.36
Subjective sleep quality	0.06 [-0.01;0.12]	0.09
Sleep latency	-0.02 [-0.09;0.05]	0.51
Sleep duration	0.03 [-0.05;0.12]	0.43
Sleep efficency	0.03 [-0.12;0.18]	0.64
Sleep disturbance	-0.001 [-0.06;0.06]	0.99
Use of sleep medication	0.06 [-0.04;0.16]	0.22
Daytime dysfunction	-0.01 [-0.09;0.06]	0.69
APS		
All Items	-0.06 [-0.59;0.47]	0.82
SCI		
All Items	-0.20 [-0.84;0.42]	0.51
FIRST		
All Items	-0.17 [-0.75;0.41]	0.55
ISI		
All Items	0.12 [-0.39;0.63]	0.64
SHI		
All Items	0.13 [-0.28;0.55]	0.52

Supplementary Figure

Supplementary Figure S1. Plot of the mean and standard deviation by time, i.e., 1=pre, 2=post, for each group, i.e., treated (blue line) and control (red line).

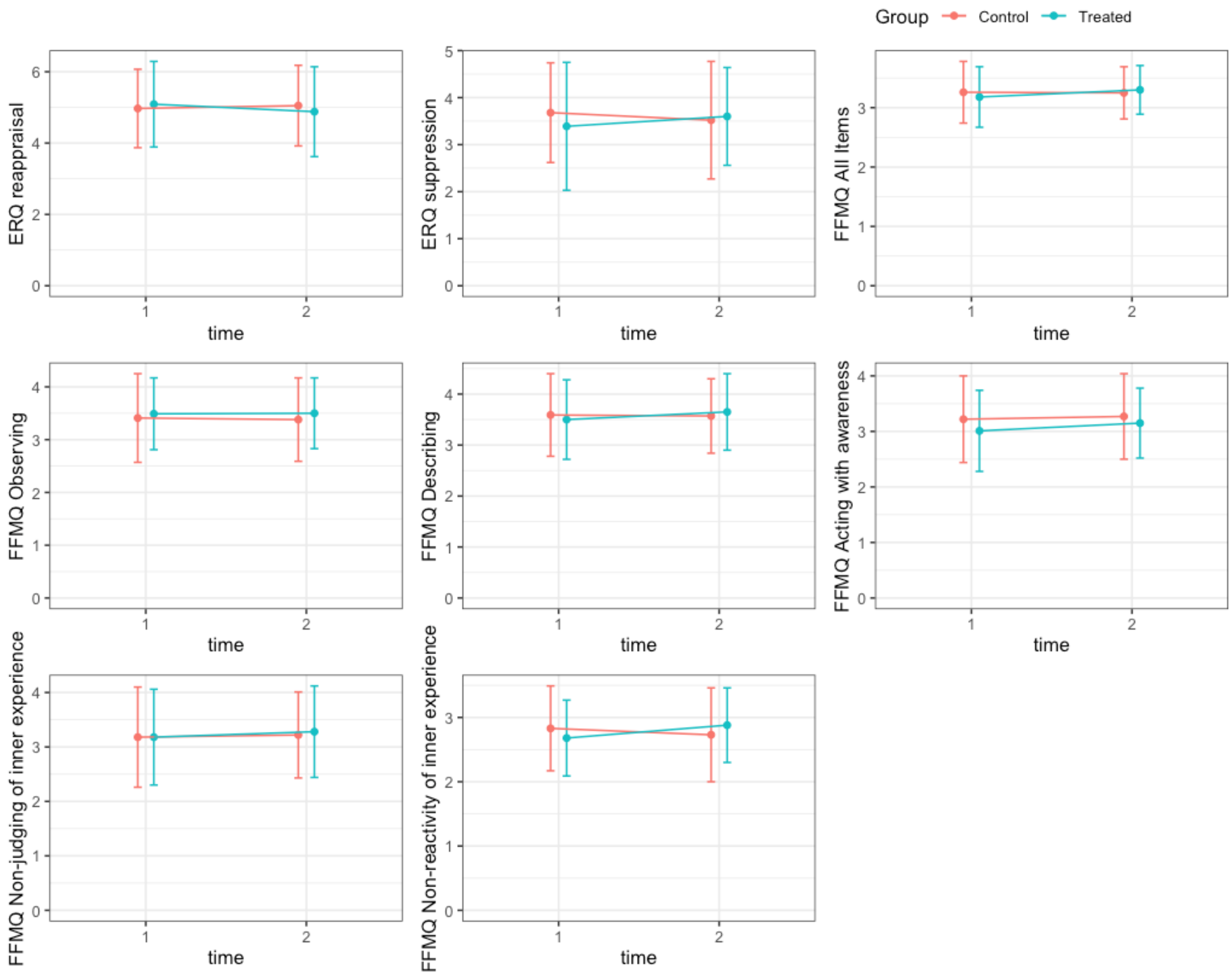

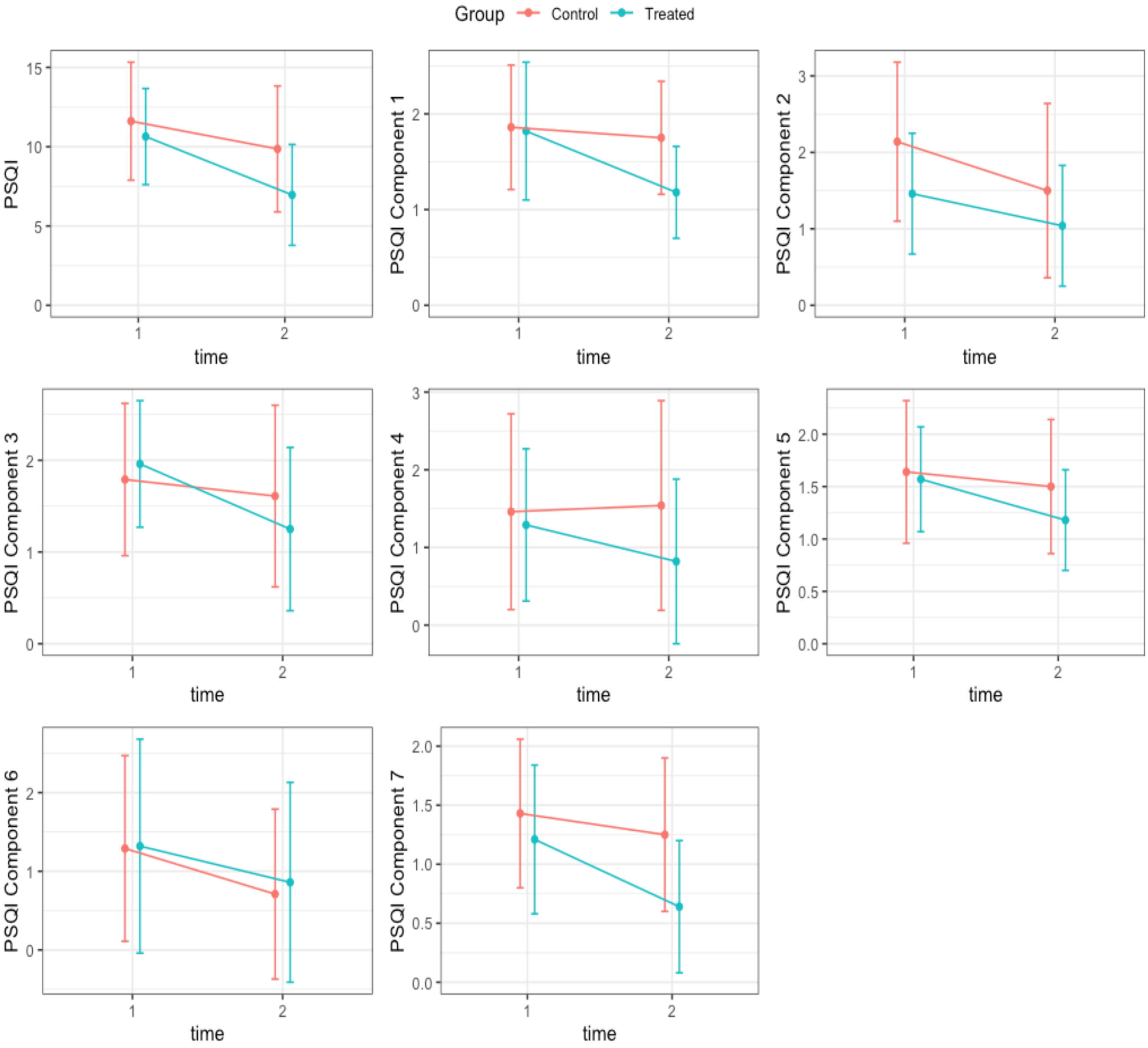
Group
Control
Treated
PSQI
PSQI Component 1
PSQI Component 2
PSQI Component 3
PSQI Component 4
PSQI Component 5
PSQI Component 6
PSQI Component 7
time

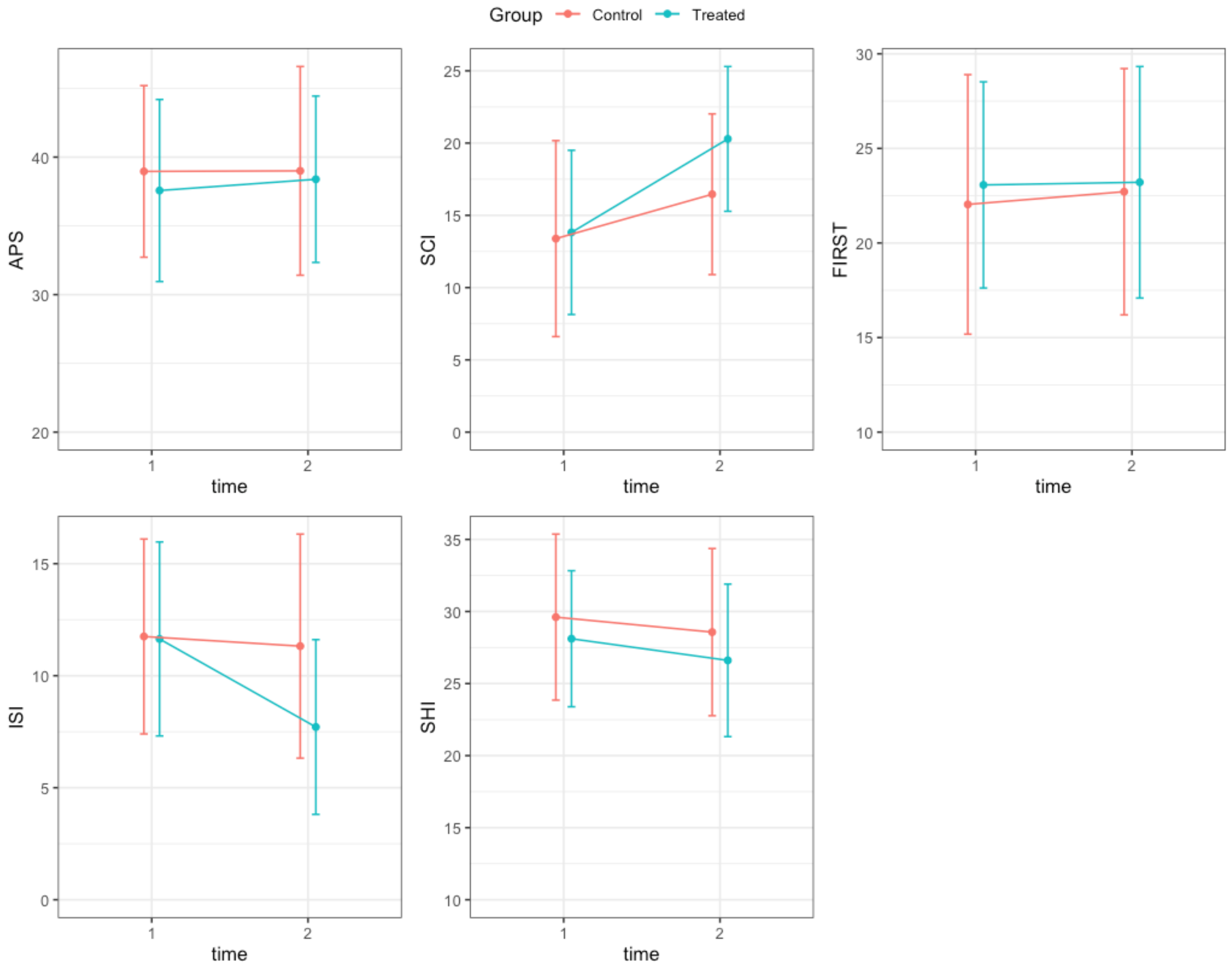
Group
Control
Treated
APS
SCI
FIRST
ISI
SHI
time
